# Case report of severe Cushing’s syndrome in medullary thyroid cancer complicated by functional diabetes insipidus, aortic dissection, jejunal intussusception, and paraneoplastic dysautonomia: remission with sorafenib without reduction in cortisol concentration

**DOI:** 10.1186/s12885-015-1620-3

**Published:** 2015-09-09

**Authors:** Muhammad M. Hammami, Najla Duaiji, Ghazi Mutairi, Sabah Aklabi, Nasser Qattan, Mohei El-Din M. Abouzied, Mohamed W. Sous

**Affiliations:** 1 Departments of Medicine, King Faisal Specialist Hospital and Research Centre, Riyadh, Saudi Arabia; 2 Departments of Clinical Studies and Empirical Ethics, King Faisal Specialist Hospital and Research Centre, P O Box # 3354 (MBC 03), Riyadh, 11211 Saudi Arabia; 3 Departments of Radiology, King Faisal Specialist Hospital and Research Centre, Riyadh, Saudi Arabia; 4 Departments of Neurosciences, King Faisal Specialist Hospital and Research Centre, Riyadh, Saudi Arabia

## Abstract

**Background:**

Normalization of cortisol concentration by multikinase inhibitors have been reported in three patients with medullary thyroid cancer-related Cushing’s syndrome. Aortic dissection has been reported in three patients with Cushing’s syndrome. Diabetes insipidus without intrasellar metastasis, intestinal intussusception, and paraneoplastic dysautonomia have not been reported in medullary thyroid cancer.

**Case presentation:**

An adult male with metastatic medullary thyroid cancer presented with hyperglycemia, hypernatremia, hypokalemia, hypertension, acne-like rash, and diabetes insipidus (urine volume >8 L/d, osmolality 190 mOsm/kg). Serum cortisol, adrenocorticoitropic hormone, dehydroepiandrostenedione sulfate, and urinary free cortisol were elevated 8, 20, 4.4, and 340 folds, respectively. Pituitary imaging was normal. Computed tomography scan revealed jejunal intussusception and incidental abdominal aortic dissection. Sorafenib treatment was associated with Cushing’s syndrome remission, elevated progesterone (>10 fold), normalization of dehydroepiandrostenedione sulfate, but persistently elevated cortisol concentration. Newly-developed proximal lower limb weakness and decreased salivation were associated with elevated ganglionic neuronal acetylcholine receptor (alpha-3) and borderline P/Q type calcium channel antibodies.

**Conclusion:**

Extreme cortisol concentration may have contributed to aortic dissection and suppressed antidiuretic hormone secretion; which combined with hypokalemia due cortisol activation of mineralocorticoid receptors, manifested as diabetes insipidus. This is the first report of paraneoplastic dysautonomia and jejunal intussusception in medullary thyroid cancer, they may be related to medullary thyroid cancer’s neuroendocrine origin and metastasis, respectively. Remission of Cushing’s syndrome without measurable reduction in cortisol concentration suggests a novel cortisol-independent mechanism of action or assay cross-reactivity. Normalization of dehydroepiandrostenedione sulfate and elevation of progesterone suggest inhibition of 17-hydroxylase and 21-hydroxylase activities by sorafenib.

## Background

Cushing’s syndrome (CS) refers to signs and symptoms caused by excessive glucocorticoids action through glucocorticoid and occasionally mineralocorticoid receptors. Ectopic adrenocorticotropic hormone (ACTH) secretion accounts for < 10 % of CS; [1] up to 7.5 % of which are due to medullary thyroid cancer (MTC) [2, 3].

Diabetes insipidus (DI) has been reported in two patients with ectopic CS and sellar metastasis [4, 5]. Although glucocorticoids can suppress antidiuretic hormone (ADH) secretion, DI has not been reported in CS without sellar lesions, possibly due to compensatory renal mechanisms [6].

MTC arises from neuroendocrine calcitonin-producing parafollicular C cells of the thyroid gland; it ectopically secrets ACTH in about 0.6 % of patients [7]. Three case reports described successful treatment of ectopic CS in MTC patients with multikinase inhibitors; in all cases, CS remission was associated with reduction in cortisol concentration [8–10].

Cardiovascular complications are rather common in CS; however, aortic dissection has been reported in only three cases [11]. Intestinal intussusception is rare in adults and is secondary to a pathological condition in 90 % of cases [12]. It has not been reported in MTC patients. Paraneoplastic dysautonomia has been associated with neuroendocrine tumors other than MTC [13].

We report a case of severe CS in a patient with metastatic MTC, which was complicated by DI without sellar lesion, silent aortic dissection, jejunal intussusception, and dysautonomia. Interestingly, sorafenib was associated with remission of CS without measurable reduction in cortisol concentration.

## Case presentation

### Case report

A 30-year old male with MTC presented with 3-week history of severe polyuria, nocturia, polydipsia, salty taste, skin rash, insomnia, and delusion. He denied vomiting, diarrhea, pain, dyspnea, cough, and fever.

He was referred about 4.5 years earlier after having total thyroidectomy that showed multifocal MTC with cervical lymph nodes metastasis. His past medical and surgical history was otherwise unremarkable. He had no family history of MTC, other tumors, or consanguinity, and negative screening for pheochromocytoma, hyerparathyroidism, and germline RET (rearranged during transfection) oncogene mutation. Calcitonin and carcinoembryonic antigen (CEA) were >5850 pmol/L (normal, <5.5) and 506 μg/L (normal, <4.3), respectively. Over the following year, he underwent bilateral neck dissection for extensive regional lymph node metastasis followed by external radiation. Computed tomography (CT) scan showed normal liver and bilateral pulmonary metastases. Calcitonin and CEA decreased to 1430 pmol/L and 287 μg/L and then increased to 5290 pmol/L and 544 μg/L, respectively, 4 months prior to admission. One year before admission, random glucose was 5.22 mmol/L.

On admission (day one), he was afebrile and appeared severely dehydrated. Pulse was 125/min, blood pressure 145/90 mmHg (previous readings, 100–110/65–75), and body mass index 20.9 kg/m^2^. He had multiple facial erythematous papules and few pustules but no moon face, centripetal obesity, supraclavicular fullness, cervical fat pad, striae, easy bruising, or stigmata of chronic liver disease except for non-tender hepatomegaly. He had normal muscle power and deep tendon reflexes. White blood cell (WBC) count was 21.7×10^9^/L (80 % neutrophils), creatinine 52 μmol/L (normal, <115), potassium 2.1 mmol/L (normal, 3.5–5.0), sodium 148 mmol/L (normal, 135–147), CO_2_ 27 mmol/L (normal, 22–31), glucose 25.7 mmol/L, albumin 35 g/L (normal, 32–48), total bilirubin 12 μmol/L (normal, <21), alanine aminotransaminase (ALT) 199 U/L (normal, 10–45), aspartate aminotransferase (AST) 117 U/L (normal, 10–45), alkaline phosphatase 216 U/L (normal, 40–135), venous blood pH 7.49 (normal, 7.30–7.40), CEA 3643 μg/L, calcitonin 1800 pmol/L, and glycated hemoglobin 0.07 (normal, <0.065). Insulin, intravenous fluid and potassium replacement, enoxaparin 40 mg/d, and topical acne treatment were started, and levothyroxine 150 μg, vitamin D3 2000 unit, and calcium carbonate 1200 mg daily were continued.

Urine output was consistently >8 L/d, while plasma and urine osmolality were 296/310 (normal, 275–300) and 189/190 mOsm/kg, respectively; and sodium, potassium, glucose, and urea 154/148, 2.9/2.8, 21.5/14.5, and 2.6/2.3 (normal, 4.2–4.2) mmol/L, respectively. Intranasal desmopressin (DDAVP) 40 μg/d reduced urine output to 4.85 L/d (sodium, potassium, and glucose were 149, 3.5, and 12.1 mmol/L, respectively).

On day 5, cortisol and ACTH were 3782 nmol/L (normal, 171–536) and 872 ng/L (normal, 5–60; to convert to pmol/L multiply by 0.22), respectively, urinary free cortisol 129,204 nmo (normal, 100–379), dehydroepiandrostenedione sulfate (DHEAS) 50.8 μmol/L (normal, 4.42–11.50), dehydroepiandrostenedione (DHEA) 12 ng/mL (Mayo Medical Laboratories (MML), Mayo Clinic, Rochester, MN; normal, <13; to convert to nmol/L multiply by 3.47), androstendione >35 nmol/L (normal, 2.4-12.6), renin 19.6 mU/L (normal, 4.4–46.1), and aldosterone < 8 ng/dL (MML; normal, < 21; to convert to nmol/L multiply by 0.277). 8-mg dexamethasone suppression test showed a paradoxical increase in cortisol to 4279 nmol/L. Urinary potassium was >100 mmol/d with plasma potassium of 3.3 mmol/L.

**Fig. 1:**
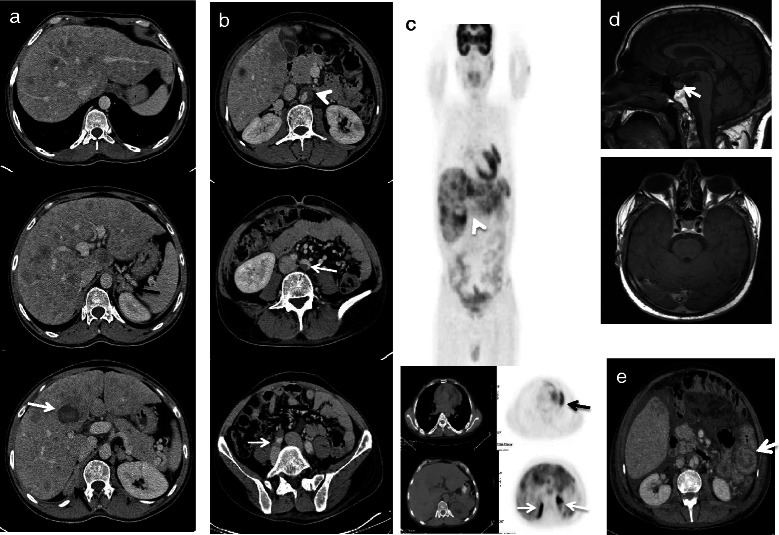
Major radiological findings. **a** Trans axial images of enhanced computed tomography (CT) of the abdomen showing: hypodense liver lesions in both lobes (the largest is located in segment IV adjacent to porta hepatis, white arrow), and bilateral adrenal hyperplasia (black arrows). **b** Aortic dissection below the level of renal arteries (white arrow head) extending to the left and right common iliac arteries (white arrows). **c** FDG PET-CT scan showing multiple hyper metabolic liver lesions (white arrow head), lung metastasis involving the left paracardiac area (black arrow), and diffuse bilateral hyper metabolic activity within the adrenal gland (white arrows). **d** Sagittal and axial (post contrast) T1 weighted MRI images showing normal pituitary gland. **e** Trans axial image of enhanced computed CT of the abdomen showing jeujenal intussusception

CT scan showed 1.8 cm pretracheal lymph node, bilateral hilar lymphadenopathy, 1.4 cm left paracardiac lymph node, progression of lung metastases bilaterally (largest 3 cm), new liver metastases (largest 5x5 cm with central necrosis and hemorrhage), enlarged adrenal glands (Fig. 1a), and short segment of dissection in abdominal aorta with thrombosed false lumen (Fig. 1b). Aortic dissection was managed conservatively by enoxaparin and blood pressure control. Fluorodeoxyglucose (FDG) positron emission tomography (PET)-CT scan showed multiple hypermetabolic liver and lung lesions and diffuse bilateral hypermetabolic adrenal activity (Fig. 1c). Pituitary magnetic resonance imaging (MRI) was normal (Fig. 1d).

**Fig. 2:**
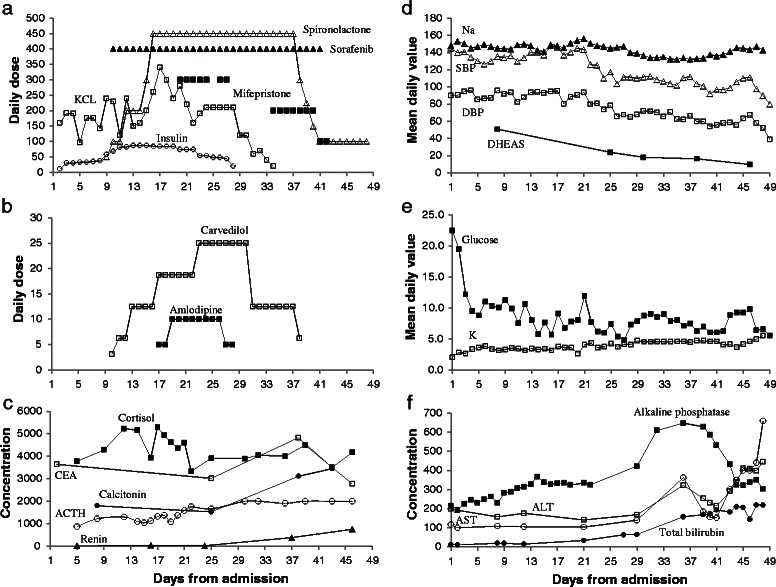
Main treatments and clinical and laboratory findings over the course of hospitalization. **a** Daily doses of spironolactone (mg, open triangles), sorafenib (mg, closed triangles), mifepristone (mg, closed squares), potassium chloride (mmol potassium, open squares), and insulin (units, open circles). **b** Daily doses of carvedilol (mg, open squares) and amlodipine (mg, closed squares). **c** Concentrations of cortisol (nmol/L, closed squares), carcinoembryonic antigen (CEA, μg/L, open squares), calcitonin (pmol/L, closed circles), adrenocorticoitropic hormone (ACTH, ng/L, open circles; values greater than 2000 ng/dL are reported as 2000 ng/L; multiply by 0.22 to convert to pmol/L), and renin (mU/L, closed triangles). Aldosterone was < 8 and < 4 ng/L (multiply by 0.277 to convert to nmol/L) on days 5 and 36, respectively. **d** Mean daily concentration of sodium (mmol/L, closed triangles), mean daily measurements of systolic (mmHg, open triangles) and diastolic blood pressure (mmHg, open squares), and concentration of dehydroepiandrosterone sulfate (DHEAS, closed squares, μmol/L). Dehydroepiandrosterone (DHEA) was 12 and 3.9 ng/mL on days 8 and 25, respectively (multiply by 3.47 to convert to nmol/L). Progesterone was 40.4 and 42.2 nmol/L on days 40 and 43, respectively. 17-hydroxyprogestrone was 6.2 nmol/L on day 40. **e** Mean daily concentration of glucose (mmol/L, closed squares) and potassium (mmol/L, open squares). **f** Concentrations of alkaline phosphatase (U/L, closed squares), alanine aminotransaminase (ALT, U/L, open squares), aspartate aminotransferase (AST, U/L, open circles), and total bilirubin (μmol/L, closed circles). Prothrombin time was 15.4, 17.4, 22.1, 31.5, and 37.6 s on days 16, 27, 36, 43, 48, respectively. Albumin was 35, 32, and 19.7 g/L on days 1, 21, and 41, respectively

Escalating doses of insulin (up to 87 U/d), spironolactone (up to 450 mg/d), oral and intravenous potassium (up to 340 mmol/d), amlodipine (up 10 mg/d), and carvedilol (up to 25 mg/d) were required to control CS (Fig. 2a&b). Adrenalectomy was not offered because of aortic dissection/thrombosis necessitating anticoagulation. Ketoconazole and mitotane were not used because of liver dysfunction, and metyrapone because of severe hypokalemia. Sorafinib 400 mg twice daily was started on day 10 (vandetanib and cabozantinib were unavailable). DDAVP was stopped because it may stimulate ectopic ACTH secretion; four days later, urine output increased to 7.8 L/d with ADH of 3.3 pg/mL (MML; normal, 0.0-6.9; to convert to pmol/L multiply by 0.926), plasma and urine osmolality of 311 and 522 mOsm/kg, respectively, and sodium, potassium, and glucose of 149, 3.5, and 14.8 mmol/L, respectively.

On day 15, he developed dyspnea, cough, mild hemoptysis, and oxygen desaturation without fever. Echocardiography was normal. CT scan showed no evidence of pulmonary embolism and new diffuse bronchial-wall thickening and multiple patchy ground-glass opacities. WBC was 13.6×10^9^ (94 % neutrophils). Sputum culture was positive for streptococcus pneumonia. Procalcitonin concentration was 110 ng/mL (normal, <0.5; severe bacterial infection, >2.0). He was treated vancomycin followed by ceftriaxone (till day 42). ^99m^Tc-Octreotide scan revealed that the liver and lung lesions were not octreotide avid.

On day 20, because of no measurable changes in ACTH and cortisol concentrations and development of proximal lower limb weakness (could get up from chair unassisted with difficulty), mifepristone 300 mg/d was started. At that time, blood pressure was 141/89 mmHg and sodium, potassium, and glucose 151, 3.6, and 7.8 mmol/L, respectively.

On day 25, CEA concentration decreased to 3019 μg/L, calcitonin to 1530 pmol/l (Fig. 2c), DHEAS to 23.96 μmol/L (Fig. 2d), and DHEA to 3.9 ng/ml (Fig. 2c). Further, blood pressure normalized to 118/78 mmHg and sodium, potassium, and glucose to 145, 4.3, and 7.5 mmol/L, respectively (Fig. 2d&e). However, cortisol and ACTH did not decrease (3911 nmol/L, 1664 ng/L, respectively) (Fig. 2c), and androstenedione continued to be >35 nmol/L. Renin was 22.1 mU/L.

On day 27, he complained of intermittent colicky abdominal pain increasing in severity and frequency, loss of appetite, and generalized fatigability in association with low normal blood pressure and glucose despite reducing insulin and amlodipine doses. Procalcitonin concentration was 281 ng/ml. Adrenal insufficiency was suspected, mifepristone discontinued, and intravenous dexamethasone given for 5 days (initially 8 mg/d) without remarkable improvement. The abdominal pain was treated with fentanyl patch supplemented with intravenous morphine. CT scan showed bilateral pleural effusion, reduction in the left paracardiac lymph node to 1 cm with central necrosis, mild reduction in the size of liver metastases, ascites, patent portal vein, no evidence of biliary dilatation, no change in the appearance of aortic dissection or adrenal glands, and jejuno-jejunal intussusception (Fig. 1e) with fecal loading without evidence of bowel obstruction.

On day 34, proximal lower limb weakness progressed to the degree that he was bed-bound, despite remarkable control of CS manifestations (Fig. 2c-e), and was associated with loss of deep tendon reflexes. There was no muscle pain, tenderness, atrophy, or post-exercise facilitation. Electrolytes were normal. The unexplained weakness together with the development of difficulty in swallowing due to reduced salivation (requiring saliva substitute every four hours, modified barium swallow evaluation was normal), difficulty in urination, constipation, and rather unexplained postural tachycardia prompted investigations for neurological autoimmunity. A paraneoplastic autoantibody screen revealed elevated neuronal ganglionic acetylcholine (alpha-3) autoantibodies of 0.12 nmol/L and borderline P/Q type calcium channel autoantibodies of 0.02 nmol/L (MML; normal, <0.02), consistent with autoimmune dysautonomia. Nerve conduction and repetitive nerve stimulation studies were unremarkable (23 % amplitude increment with 30 stimuli/s). Electromyography of the right anterior tibial and vastus medialis muscles showed reduction in mean amplitude (0.13 and 0.15 mV, normal 0.22 and 0.23 mV, respectively) and duration (8.5 and 8.8 ms, normal 13.1 and 10.9 ms, respectively). 25 Hydroxyvitamin D was 15 nmol/L, free T4 6.4 pmol/L (normal, 12–22), thyroid stimulating hormone (TSH) 1.27 mU/L (normal, 0.27–4.20), and parathyroid hormone (PTH) 20.5 ng/L (normal, 15–65; to convert to pmol/L multiply by 0.1061).

Despite persistently elevated ACTH and cortisol, starting on day 20, insulin and potassium doses had to be tapered off to avoid hypoglycemia and hyperkalemia (Fig. 2a&e). Further, mifepristone which was restarted on day 34 at 200 mg/d had to be decreased to 100 mg/d after 7 days to be stopped 2 days later (Fig. 2a). Similarly, starting on day 30, amlodipine and carvedilol had to be tapered off to avoid hypotension (Fig. 2b). Spironolactone was reduced gradually to 100 mg/d on day 41 (continued for ascites and lower limb edema) (Fig. 2a). By day 31, urine output normalized and he had mild hyponatremia. On day 37, ADH was 3.9 pg/ml, plasma and urine osmolality 284 and 552 mOsm/kg, respectively, and sodium, potassium, glucose, and urea 133, 4.8, 5.33, and 5.7 nmol/L, respectively. Renin increased to 385 and 757 mU/L on days 37 and 46, respectively (Fig. 2c). DHEAS decreased to 9.85 μmol/L on day 46 (Fig. 2d). On day 40, progesterone and 17-hydroxyprogestrone concentrations were 40.4 nmol/L (normal for adult males, 0.7–4.3) and 6.2 nmol/L, respectively. On day 43 progesterone was 42.2 nmol/L. To verify the persistently elevated cortisol, a split sample was sent to another laboratory (competitive binding immunoenzymatic assay, MML). Cortisol was 4170 nmol/L in our laboratory and 114 μg/dL (normal, 7–25; to convert to nmol/L multiply by 27.6) in the other, representing 7.8 vs. 4.6 fold increase over upper limits, respectively.

Liver function tests deteriorated sequentially, starting with an increase in alkaline phosphatase and total (direct) bilirubin followed by a decrease in albumin, prolongation of prothrombin time, and an increase in ALT and AST (Fig. 2f). Screening for hepatitis A, B, and C was negative. Liver US/Doppler showed enlarged liver studded with metastatic disease, patent main portal vein with sluggish flow, flow reversal within the left portal and splenic veins, and no biliary dilatation.

On day 42, he had desaturation, sputum grew pseudomonas, and procalcitonin was >500 ng/ml. Sorafenib and mifepristone were stopped and intravenous dexamethasone was given. Liver function tests continued to deteriorate and hemoglobin dropped to 87 g/L. He died on day 49 due to cardiopulmonary arrest despite intravenous antibiotics and fresh frozen plasma, red blood cell, and albumin infusions.

Laboratory investigations were performed by our Department of Pathology and Laboratory Medicine except as indicated. Cortisol, ACTH, and DHEAS were measured by electrochemiluminescence immunoassay (Elecsys cortisol, ACTH, DHEAS kits, respectively, Roche Diagnostics, Indianapolis, IN) on cobas e analyzer following manufacturer’s specifications. Androstenedione was measured by solid-phase enzyme-labeled chemiluminescence immunoassay (DPC Immulite andorostenedione kit, Diagnostic Products Corporation, Los Angeles, CA) on DPC automated immunoassay system following manufacturer’s specifications.

## Discussion

The unique features of this unfortunate case of sporadic MTC and ectopic ACTH CS include: 1) exceedingly elevated urinary free cortisol, 2) reversible combined neurogenic and nephrogenic DI, 3) CS remission with sorafenib without measurable reduction in cortisol concentration, 4) abdominal aortic dissection, 5) jejuno-jejunal intussusception, 6) development of autoantibodies against neuronal ganglionic acetylcholine receptor, and 7) development of acute liver failure.

CS refers to signs and symptoms caused by excessive glucocorticoids action through glucocorticoid and occasionally mineralocorticoid receptors. Ectopic CS occurs in 0.6 % of MTC patients, usually with advanced disease [7]. As expected, our patient had liver and lung metastasis and markedly elevated tumor markers. However, his exceedingly high serum cortisol and urinary free cortisol were unusual. Further, despite florid derangements related to activation of mineralocorticoid and androgen receptors, hyperglycemia was the only glucocorticoid receptor-related abnormality. Having normoglycemia prior to admission and normal BMI and mildly elevated glycated hemoglobin on admission, indicated that the hyperglycemia was CS-induced and suggested that the other glucocorticoid receptor-related manifestations of CS may take longer time to develop. Severe hypokalemia and hyperkaliuria were likely due to activation of mineralocorticoid receptors by elevated cortisol that evaded inactivation by 11-beta-hydroxysteroid dehydrogenase.

The polyuria in our patient was not explained by hyperglycemia as it persisted after its correction and was associated with low urine osmolality. Further, the degree and persistence of polyuria were more than expected from the degree of hypokalemia, suggesting a neurogenic DI component. This was confirmed by a partial response to DDAVP, recurrence after stopping DDAVP, and an ADH that was inappropriately in the low normal range. DI was reported in two MTC patients without CS [4, 5] and in two patients with small-cell lung cancer and CS [14, 15]. In all cases there was intrasellar metastasis. Pituitary MRI in our patient was normal, suggesting functional neurogenic DI. As little as 30 mg prednisolone for 5 days inhibited osmotically-stimulated ADH secretion, however, urine osmolar concentration was unaffected; perhaps due to ADH-independent renal compensatory mechanisms [6]. We postulate that elevated cortisol in our patient caused ADH suppression and hypokalemia prevented the proposed compensatory mechanisms. Interestingly, after blocking cortisol action and correction of hypokalemia, urine output normalized and mild hyponatremia developed.

This is the first reported case of failure of elevated cortisol to decrease in MTC-related CS treated with multikinase inhibitors. In the two cases treated with vandetanib [8, 9] and one case treated with sorafenib [10], cortisol normalized within a week. Nevertheless, CS was reversed in our patient, necessitating discontinuation of the previously-required, large doses of potassium, spironolactone, antihypertensives, and insulin. Although mifepristone may have played a role in diabetes mellitus remission, it could not explain remission of hypertension and hypokalemia, its dose was relatively small, and diabetes mellitus remission persisted despite its withdrawal.

The mechanisms underlying reduction of cortisol concentration in this setting may be multifactorial. In one case, vandetanib resulted in simultaneous decrease in cortisol and calcitonin and a blunted response of ACTH to DDAVP without reduction in tumor size, suggesting a direct antisecretory action [8]. In another case, sorafenib reduced cortisol and ACTH with modest reduction in calcitonin and CEA, suggesting a selective inhibition of ACTH and cortisol secretion; reduction in POMC mRNA expression through MAPK pathway inhibition, ACTH action down-regulation by downstream signaling pathway inhibition, and adrenal ischemia were postulated [10]. In the third case, vandetanib resulted in sustained (26 months) normalization of cortisol and ACTH. However, a mild re-increase in ACTH without an increase in cortisol occurred while on vandetanib [9]. In our patient, there was a remarkable decrease in DHEAS and elevation in progesterone, suggesting a direct adrenal action of sorafenib, namely, 21-hydroxylase and 17-hydroxylase inhibition. Progesterone exhibits the same affinity as aldosterone for the human mineralocorticoid receptor, acting as antagonist [16]. It has antagonistic activity at the glucocorticoid receptor especially with reduced receptors number [17]. Despite >10 fold increase, progesterone would not effectively compete with prevailing cortisol in our patient, since both are equally protein-bound [16]. Nevertheless, we could not exclude a role of progesterone in CS remission because of the possibility of spuriously high cortisol concentration. For example, 21-deoxycortisol, which is elevated by 21-hydroxylase inhibition, had 45 % cross-reactivity in our cortisol immunoassay [18]. Further, cortisol concentration was increased 7.8 fold over upper normal limit in our immunoassay compared to 4.6 fold in another immunoassay. Much to our regret, we did not measure cortisol concentration by a more specific liquid chromatography assay or 21-deoxycortisol concentration. Finally, sorafenib was shown to down regulate wild type and c-terminally truncated (lacking ligand binding domain) androgen receptors in prostate cancer cells and to inhibit their signaling [19]. We postulate a similar action of sorafenib on mineralocorticoid and glucocorticoid receptors in our patient. It is of note that insulin-independent hypoglycemia was induced by sorafenib in a patient with hemangiopericytoma and was responsive to glucocorticoid treatment [20].

Aortic dissection has been reported in 3 men with CS, two with adrenal adenoma and one with pituitary adenoma; all three presented with dissection-related symptoms [11]. In our patient, aortic dissection was discovered accidently. The prevalence of silent aortic dissection in CS is not known as most cases of CS are due to exogenous glucocorticoids or Cushing’s disease and abdominal imaging is not performed. A causal relationship between elevated cortisol and aortic dissection can be postulated; cortisol could increase blood vessels fragility through negative effects on collagen formation and connective tissue strength. Acute elevation in blood pressure is another potentially contributing factor.

Intestinal intussusception in adults is rare accounting for 5 % of all intussusceptions; most cases are secondary to lead lesions requiring surgical intervention; and presenting symptoms are usually chronic and nonspecific [12]. To the best of our knowledge, intestinal intussusception has not been reported in MTC, nor has intestinal metastasis. We postulate that the intussusception was the cause of the intermittent abdominal pain in our patient and that it was likely due to metastasis, however, functional intussusception cannot be excluded as a mass was not visualized.

Lambert-Eaton syndrome (LES), a rare neuromuscular transmission disorder, characterized by proximal muscle weakness, depressed deep tendon reflexes, post-tetanic potentiation, and autonomic dysfunction, is caused by autoimmunity against P/Q voltage-gated calcium channel [21, 22]. It is most commonly associated with small-cell lung cancer but has not been reported in MTC [22]. Our patient presented with normal power despite having profound hypokalemia and hypercortisolemia, started to have mild weakness 20 days later when hypokalemia was corrected, and his weakness rapidly progressed to the degree that he became bed-bound despite mifepristone treatment. Electrolytes and TSH were normal. 25- Hydroxyvitamin D concentration was low, however, this was likely due to low binding proteins since PTH and corrected calcium were normal, and he had been on 2000 U of vitamin D daily. Absence of deep tendon reflexes, markedly decreased salivation, and postural tachycardia suggested LES and autoimmune dysautonomia. We could not confirm LES diagnosis since repetitive stimulation testing was negative and P/Q voltage-gated calcium channel antibodies were only upper normal. However, electromyography revealed motor unit potentials of short duration and decreased amplitude as described in LES [23] and neuronal ganglionic acetylcholine receptor autoantibodies, the only proven effector of autoimmune dysautonomia [13], were markedly elevated; which to our knowledge has not been described in MTC.

Another challenge we encountered in managing the patient was related to using procalcitonin to diagnose bacterial infection. Elevated procalcitonin, in general, indicates bacterial infection [24]. However, procalcitonin was useless in our patient since it is elevated in MTC patients [25] and may be elevated in adrenal crisis [26].

About 50 % of sporadic MTC carry RET gene somatic mutations, and RAS mutations are observed in about 50 % of RET-negative tumors [27]. The multikinase inhibitors, vandetanib and cabozantinib, were recently approved to treat symptomatic or progressive MTC [10]. Sorafenib was investigated in Phase II trials [28] and was successfully used to treat CS in MTC [10]. Since vandetanib and cabozantinib were not available in our institution, we treated our patient with sorafenib. There was an initial 15 % decrease in calcitonin and CEA and regression in some of the liver and lymph node metastasis within 17 days of treatment, however, calcitonin and CEA quickly rebounded and liver metastasis progressed.

Acute liver failure, characterized by the development of jaundice, coagulopathy, and hepatic encephalopathy within 8 weeks in the absence of preexisting liver disease, was reported in a patient with liver metastasis from MTC [29]. Liver functions in our patient deteriorated rapidly over 7 weeks, while screening tests for hepatitis A, B, and C were negative. Although iatrogenic liver injury due to sorafenib could not be excluded, it is likely that progression of liver metastasis was the culprit as suggested by the results of hepatic ultrasound/Doppler results. Further, mild liver test abnormalities have been reported in <1 % of sorafenib treated patients, and severe acute hepatitis is very rare, usually of the hepatocellular type, and is associated with high fever and rash; [30] which was not the case in our patient.

## Conclusions

Extremely elevated cortisol may have contributed to aortic dissection, due to interference with collagen formation, and suppressed ADH secretion; which combined with hypokalemia due cortisol activation of the mineralocorticoid receptors, manifested as reversible combined neurogenic and nephrogenic DI. Paraneoplastic dysautonomia and jejunal intussusception have not been reported in MTC, they may be related to MTC’s neuroendocrine origin and metastasis, respectively. Remission of CS with sorafenib without measurable reduction in cortisol concentration suggests a novel cortisol-independent mechanism of action, such as down regulation of glucocorticoid and mineralocorticoid receptors or inhibition of their signaling. Alternatively, normalization of DHEAS and elevation of progesterone concentration suggest inhibition of 17-hydroxylase and 21-hydroxylase activities. The later could result in elevated 21-deoxycortisol that may be spuriously measured as cortisol in immunoassays. In addition, progesterone could block glucocorticoid and mineralocorticoid receptors depending on actual cortisol concentration. Further studies are required to explore potential effects of multikinase inhibitors on adrenal steroids synthesis and action, the prevalence of silent aortic dissection in CS, and the prevalence of paraneoplastic dyasautonomia in MTC.

## Consent

Written informed consent was obtained from the patient next of kin for publication of this Case report and any accompanying images. A copy of the written consent is available for review by the Editor of this journal.

## Abbreviations

CS: Cushing’s syndrome
ACTH: adrenocorticotropic hormone
MTC: Medullary thyroid cancer
DI: Diabetes insipidus
ADH: Antidiuretic hormone
RET: Rearranged during transfection
CEA: Carcinoembryonic antigen
CT: Computed tomography
WBC: White blood cell
ALT: Alanine aminotransaminase
AST: Aspartate aminotransferase
DDAVP: Desmoprssin
DHEAS: Dehydroepiandrostenedione sulfate
MML: Mayo Medial Laboratories
DHEA: Dehydroepiandrostenedione
MRI: Magnetic resonance imaging
FDG: Fluorodeoxyglucose
PET: Positron emission tomography
TSH: Thyroid stimulating hormone
PTH: Parathyroid hormone
DPC: Diagnostic products corporation
POMC: Proopiomelanocortin
MAPK: Mitogen-activated protein kinase
LES: Lambert-Eaton syndrome
